# There is a world beyond αvβ3-integrin: Multimeric ligands for imaging of the integrin subtypes αvβ6, αvβ8, αvβ3, and α5β1 by positron emission tomography

**DOI:** 10.1186/s13550-021-00842-2

**Published:** 2021-10-12

**Authors:** Katja Steiger, Neil Gerard Quigley, Tanja Groll, Frauke Richter, Maximilian Alexander Zierke, Ambros Johannes Beer, Wilko Weichert, Markus Schwaiger, Susanne Kossatz, Johannes Notni

**Affiliations:** 1 Institut Für Pathologie Und Pathologische Anatomie, Technische Universität München, Munich, Germany; 2 Klinik Für Nuklearmedizin Und Zentralinstitut Für Translationale Krebsforschung (TranslaTUM), Klinikum Rechts Der Isar der Technischen Universität München, Munich, Germany; 3 Klinik Für Nuklearmedizin, Universitätsklinikum Ulm, Ulm, Germany; 4 Experimental Radiopharmacy, Clinic for Nuclear Medicine, University Hospital Essen, Hufelandstr. 55, 45147 Essen, Germany

**Keywords:** Gallium-68, Integrins, Positron emission tomography, Radiopharmaceuticals, Theranostics

## Abstract

**Background:**

In the context of nuclear medicine and theranostics, integrin-related research and development was, for most of the time, focused predominantly on 'RGD peptides' and the subtype αvβ3-integrin. However, there are no less than 24 known integrins, and peptides without the RGD sequence as well as non-peptidic ligands play an equally important role as selective integrin ligands. On the other hand, multimerization is a well-established method to increase the avidity of binding structures, but multimeric radiopharmaceuticals have not made their way into clinics yet. In this review, we describe how these aspects have been interwoven in the framework of the German Research Foundation's multi-group interdisciplinary funding scheme CRC 824, yielding a series of potent PET imaging agents for selective imaging of various integrin subtypes.

**Results:**

The gallium-68 chelator TRAP was utilized to elaborate symmetrical trimers of various peptidic and non-peptidic integrin ligands. Preclinical data suggested a high potential of the resulting Ga-68-tracers for PET-imaging of the integrins α5β1, αvβ8, αvβ6, and αvβ3. For the first three, we provide some additional immunohistochemistry data in human cancers, which suggest several future clinical applications. Finally, application of αvβ3- and αvβ6-integrin tracers in pancreatic carcinoma patients revealed that unlike αvβ3-targeted PET, αvβ6-integrin PET is not characterized by off-target uptake and thus, enables a substantially improved imaging of this type of cancer.

**Conclusions:**

Novel radiopharmaceuticals targeting a number of different integrins, above all, αvβ6, have proven their clinical potential and will play an increasingly important role in future theranostics.

## Introduction

Multimerization is a venerable concept, and its theoretical foundations have been established decades ago [1]. There is no general doubt about the potential benefits of combining more than one targeting moiety (receptor ligands, enzyme inhibitors, antibodies or -fragments, or others), in view of a solid body of evidence that multimers invariantly exhibit a higher avidity than monomers [1, 2]. Böhmer et al. nevertheless pointed out that in despite of the long-known, huge potential of multimers and a lot of pertinent research, such compounds have made no impact in molecular imaging beyond the in vitro or preclinical levels [1], aside from full-size antibodies which are natural dimers of targeting proteins.

A similar situation—a sound and logical concept, intense long-term research, yet very limited clinical impact—is observed for radiopharmaceuticals targeting integrins. In sharp contrast to the tremendous clinical and commercial success of prostate specific membrane antigen (PSMA) targeted radiopharmaceuticals since 2015, radiolabeled integrin ligands have not been included in healthcare schemes, although they were clinically tested about one decade earlier [3–6], and selected ones even entered clinical trials several years ago [7–9]. It is therefore not surprising that the general attitude toward integrin-targeting radiopharmaceuticals has considerably changed over time. Two decades ago, the development [10] and first successful clinical applications of the positron emission tomography (PET) radiopharmaceutical ^18^F-Galacto-RGD [3–5] caused a veritable enthusiasm and unleashed an avalanche of similar agents, which were initially celebrated as a new class of highly promising peptidic radioligands for imaging of (tumor) angiogenesis [11, 12]. These days, however, one cannot help noticing a certain fatigue or even resignation because none of the many integrin tracers, even of the respective multimers which occasionally showed superior in vivo properties [6], has become clinically relevant [2].

We argue that this sobering balance is caused by the fact that pertinent research focused predominantly on the subtype αvβ3—which is, however, only one of 24 known integrins, whose wealth of biological implications and potential applications has been widely underestimated or even disregarded in the context of radiopharmaceuticals and molecular imaging agents for a long time. This article will shed light on both aspects—integrins and multimers—and describe how the challenges of either of which have ultimately been overcome owing to continuous research within the framework of the Collaborative Research Centre 824 (CRC824), resulting in integrin targeted radiopharmaceuticals with a realistic clinical perspective.

## αvβ3-Integrin targeting radiopharmaceuticals—A critical analysis

A look on the wealth of pertinent literature reveals that the terms "integrin," "αvβ3," "RGD" and "(neo-)angiogenesis" are often closely associated. Frequently, they are even used in a synonymous manner [13], which might be a result of historical development. As early as in 1984, it was discovered that some integrins accept the peptide motif arginine–glycine–aspartate, which is abbreviated by 'RGD' in the one-letter code, as a minimal amino acid sequence for recognition of their natural ligands (extracellular matrix proteins such as fibronectin, vitronectin, and fibrinogen) [14]. 1991 saw the first report on cyclic pentapeptides containing the RGD sequence, which were capable of antagonistic binding to αvβ3-integrin with high affinity and selectivity [15]. Some peptides of this class have become extraordinarily popular in the meantime, e.g., cyclo-[RGDfK], cyclo-[RGDyK], cyclo-[RGDfE], or cyclo-[RGDf(*N*Me)V] (cilengitide, EMD 121,974). These are widely referred to as 'RGD peptides,' and a clear distinction between the different compounds is rarely made.

By 1994, Cheresh and coworkers found that αvβ3-integrin plays a major role in angiogenesis, i.e., the sprouting of new vessels from existing ones (to be distinguished from de-novo formation of vasculature, called vasculogenesis) [16]. This process is not only of fundamental importance for embryonal development, wound healing, and chronic inflammation [17], it also represents a key step in the development of solid tumors. Upon reaching a critical size of a few millimeters in diameter, their enhancing demand of nutrients and oxygen can no longer be satisfied by diffusion and thus, triggers the formation of blood vessels (a signaling cascade referred to as 'angiogenic switch') [16]. This resulted in the intriguing perspective of utilizing 'RGD peptides' to block tumor angiogenesis and, therefore, tumor growth, in analogy to anti-VEGF antibodies like bevacizumab. The career of 'RGD peptides' in nuclear medicine commenced with the idea to identify patients whose tumors express αvβ3-integrin and who, therefore, would benefit from such treatment, by noninvasive molecular imaging using radiolabeled 'RGD peptides' as tracers [11]. By and by, this seemingly simple and universally applicable concept of targeting angiogenic processes with 'RGD peptides' became a popular narrative in life sciences [18]. The scheme was utilized and adapted in many ways for directing all kinds of vehicles, for example, radiopharmaceuticals, contrast agents, fluorescent dyes, nanoparticles, micelles, and chemotherapeutics, to angiogenic sites—preferably, to tumor lesions [11, 12, 19, 20].

This notion is somewhat problematic, not because it is incorrect, but because it does not picture reality in its entirety. First, there are also integrin-independent pathways that regulate angiogenesis, such as vascular endothelial growth factor receptor 2 (VEGFR2) signaling [21]. Second, it became apparent that neither the αv-[22] nor the β3-subunit [23] (and, therefore, αvβ3) is strictly required for angiogenesis. αvβ3-integrin is furthermore found on macrophages [24] and many tumor cells [20]. The expression of αvβ3-integrin in tissues is therefore neither a necessary nor a sufficient condition for angiogenesis, and a causal relationship between these two instances does not exist [25]. Phrases like "angiogenesis imaging using RGD" [13] are therefore misleading and should be avoided. Actually, this assumed interdependency has already been widely denied in the course of preclinical evaluation of many αvβ3-integrin targeting radiopharmaceuticals. These were frequently evaluated in mice bearing subcutaneous xenografts of cell lines with a strong membranous expression of αvβ3-integrin, such as U87MG [26] or M21 [10]. Accumulation of the respective radiopharmaceuticals in such tumors is therefore not unequivocally effected by binding to αvβ3-integrin expressed by the (murine!) endothelium, but at least partly (in most instances, predominantly) by binding to the human tumor cells [27]. αvβ3-integrin imaging does therefore not allow for an assessment of angiogenic activity or vessel density of the respective tumor xenografts.

This conceptual change was consequently transferred to clinical investigations. Radiolabeled 'RGD-peptides' were frequently applied for tumor imaging, e.g., as a possible alternative to [^18^F]FDG, quietly disregarding the question whether a tracer uptake might actually be related to angiogenesis or not [2, 7]. It, however, seems to consolidate that the average αvβ3-integrin expression density on tumor cells and -endothelium is simply not sufficient to guarantee a clinical impact comparable to somatostatin receptor (SSTR)-, PSMA-, or fibroblast activating protein (FAP) targeted radiopharmaceuticals. On the way to theranostics, that is, the tandem application of nuclear imaging agents and the matching therapeutics labeled with particle emitters such as ^177^Lu, ^90^Y, or ^225^Ac, another obstacle is encountered in the form of a non-negligible physiological αvβ3-integrin expression in some organs, which inevitably causes substantial background uptakes and thus, unwanted organ doses [7]. αvβ3-integrin targeted radiopharmaceuticals have therefore not made their way toward routine clinical diagnostics and therapy of cancer. After a long period of thorough clinical testing of various agents without convincing results, it is furthermore hardly imaginable that they will ever prevail. It remains to be seen whether promising non-oncological applications are eventually emerging, such as prediction of cardiac remodeling [28], or even completely new approaches, such as diagnostics of primarily endothelial diseases like the post-COVID-19 syndrome.

## RGD or not RGD—that is the question

**Fig. 1:**
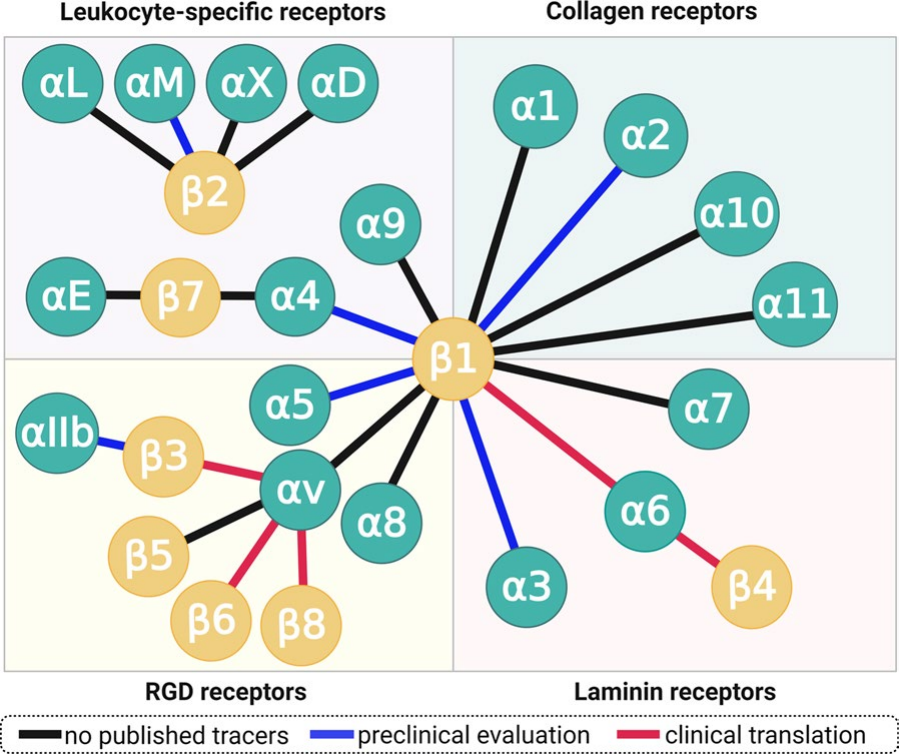
The integrin receptor family: Integrin subunits and the known dimers formed. Each connecting line represents one integrin. Although small-molecule ligands have been developed for many subtypes, radiolabeled derivatives thereof were reported only for a fraction of integrins (blue and red lines). "Clinical translation" encompasses all hitherto reported applications of such radiopharmaceuticals in humans, i.e., not only clinical trials but also single cases and small cohorts

In light of this situation, the recently cooled enthusiasm concerning integrin tracers comes as no surprise. However, a broader view on integrins is becoming more and more popular within radiopharmacy, nuclear medicine, and beyond. After all, there are no less than 24 different integrins, which are each formed by dimerization of one out of 18 α- and 8 β-subunits (Fig. 1). Eight of them recognize the RGD sequence (i.e., RGD is the primary recognition motif in their natural ligands). 'RGD peptides' can therefore be utilized to address seven integrins other than αvβ3, necessitating to re-adjust the associations made with the term 'RGD peptide.'

**Fig. 2:**
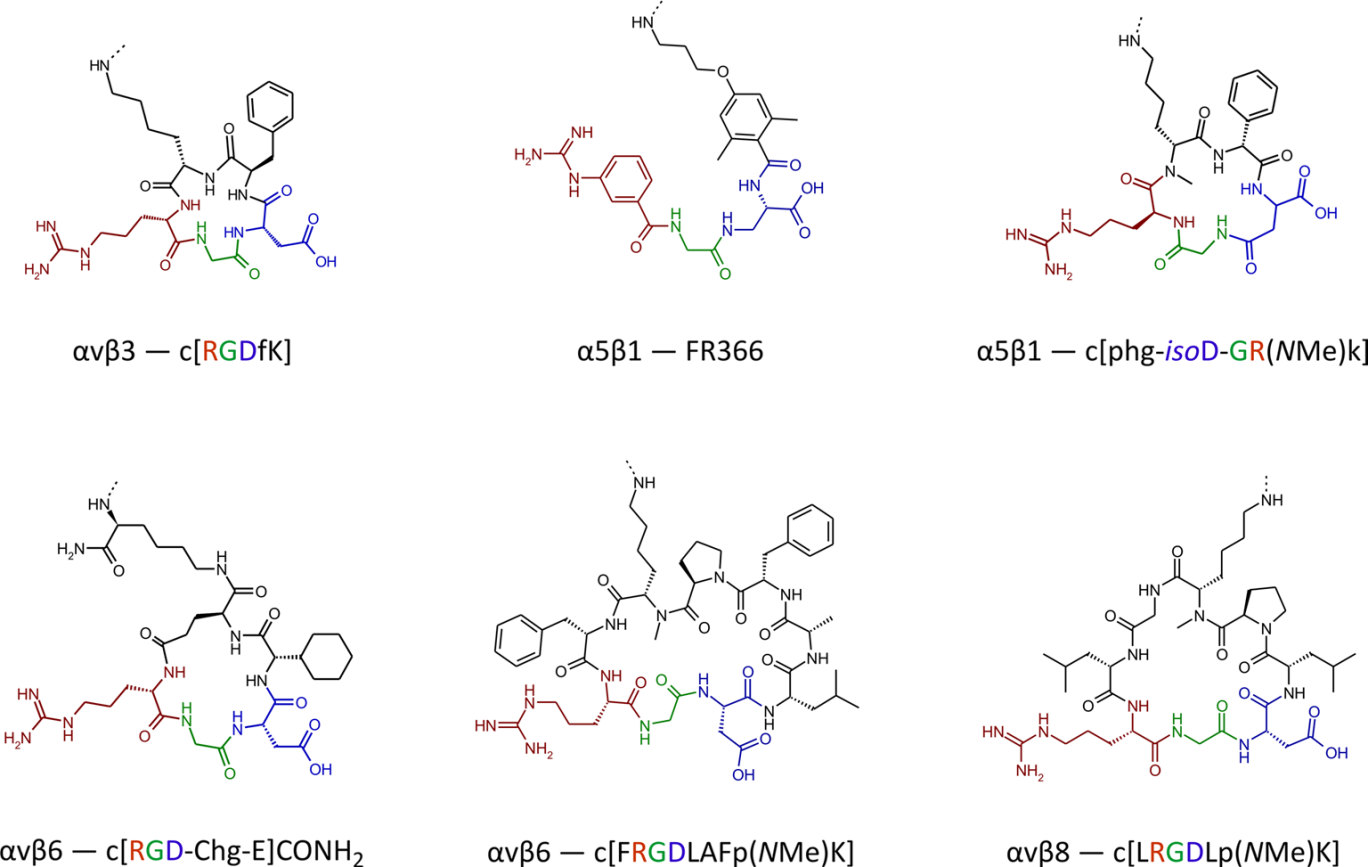
Examples of selective integrin ligands [15, 30–34] whose radiolabeled derivatives were developed and/or evaluated in the framework of CRC 824. Note that all addressed integrins (αvβ3, αvβ6, αvβ8, α5β1) belong to the class of RGD receptors (see **Fig. ****1**), but not all of the peptides contain the RGD sequence, and one ligand (FR366) is not a peptide but a peptidomimetic. The colors highlight the RGD sequence or their structural equivalents, respectively. The dashed bonds on the terminal amines indicate the conjugation sites

The short RGD sequence can be extended on both termini, resulting in linear peptides which bind equally well, or even preferably, to integrins other than αvβ3 [29]. Incorporation of the RGD motif into cyclic peptides or three-dimensional peptide knots appears to be the most promising approach, because they are generally more resistant toward enzymatic cleavage than linear peptides. The conformationally stable, three-dimensional shape of such ring or cage structures is often further rigidified by intramolecular hydrogen bonds. Such rigid structures essentially fix a certain conformation (folded, distorted, bent, or stretched) of the RGD motif, which ideally facilitates selective binding to a certain integrin whose unique binding pocket perfectly accommodates just that particular conformation [29]. Some examples for such selective ligands are shown in Fig. 2 [15, 30–34], which furthermore illustrates that ligands for RGD-binding integrins do not necessarily have to comprise the RGD amino acid sequence at all. Some organic molecules with a more or less peptide-like structure (so-called peptidomimetics) have been described, which are highly selective for integrins αvβ3 or αvβ6 [35], α5β1 [36], or αIIbβ3 (cf. tirofiban, an antiplatelet drug). Furthermore, the linear peptide RTDLDSLRT does not feature an RGD motif, but nevertheless shows a good affinity (30 nM) for the RGD-recognizing αvβ6-integrin, and furthermore a pronounced selectivity over other RGD-binding integrins (tenfold over αvβ8, > 200-fold over αvβ3, αvβ5, α5β1, and αIIbβ3) [29]. These examples demonstrate that a particular, frequently asked question—whether or not a selective ligand for a given integrin is a 'RGD peptide'—is largely irrelevant for practical application. Likewise, distinguishing between RGD-binding and other integrins unnecessarily erects mental barriers on the way to integrin-targeted theranostics and their use in personalized medicine.

## Toward a greater variety of targeted integrins

A far more important question is whether a given integrin might be a useful target, i.e., whether its expression is correlated with a relevant clinical problem [37], and whether its physiological presence in normal tissue is low enough to minimize homing of diagnostic and/or therapeutic agents to non-disease areas. The available data are frequently not sufficient for a reliable prediction, which is admittedly quite difficult for integrins. The actual density of a fully functional and activated integrin on a cell surface, which is determining its value for in vivo targeting, cannot be quantified solely on the basis of upstream biomarkers, such as mRNA concentration [39]. It has to be kept in mind that integrins are composed of two separate proteins, one α- and one β-chain, which are encoded, transcribed, and translated independently of each other. After dimerization and transport to the cell membrane, integrins require activation (i.e., a conformational change) initiated by intracellular signaling processes, enabling them to bind to their respective ligands (mostly extracellular matrix proteins) [38]. Being cell adhesion receptors, the expression of integrins is furthermore modulated to a certain extent by a cell's surroundings, such as the tumor microenvironment. Hence, the actual quantification of fully functional integrins in (malignant) human tissues, e.g., by immunohistochemistry (IHC), appears to be the most reliable source of information on expression patterns and their relevance for disease management. With more pertinent data generated for each integrin subtype, their potential as targets for diagnostics and therapeutics will become more apparent. Albeit the availability of such data is limited for some integrins, the current state of knowledge nevertheless allows to identify some promising approaches.

### αvβ6: The cancer integrin

Unlike αvβ3, αvβ6-integrin is not expressed by endothelial, but epithelial cells, and is furthermore widely absent in adult human tissues [39]. Its most important function is the activation of transforming growth factor β (TGFβ), a pleiotropic cytokine whose highly conserved isoforms TGFβ1–3 are produced by virtually all mammalian cells [40]. TGFβ is a powerful growth-inhibiting factor, and in order to control and regulate its signaling, it is secreted into the intracellular space in a latent, inactive complex with another protein called latency-associated peptide (LAP). αvβ6-integrin activates TGFβ by binding to an RGD sequence of LAP, and by transmitting an actual pulling force, the protein complex is deformed and releases TGFβ [41, 42]. Hence, the expression of αvβ6-integrin is tightly connected to diseases rooted in, or related to, altered TGFβ signaling.

The apparent most important implication of the described biochemistry is that αvβ6-integrin is a driver for invasion and metastasis of epithelial cancers (carcinomas) [43]. This is because TGFβ normally regulates tissue growth by inhibiting several proliferative signaling cascades. Carcinoma cells, however, frequently lose certain components of the respective downstream pathways, for example, p53 [44] or Smad4 [45], and become insensitive to TGFβ-induced growth inhibition. Thus, they benefit from a high TGFβ level in their surroundings, because it inhibits proliferation of the surrounding normal cells but not their own [46]. Overexpression of αvβ6-integrin therefore helps carcinomas to invade normal tissues. Consistent with this picture, the highest αvβ6 expression densities are found in infiltrative tumor margins [47].

**Fig. 3:**
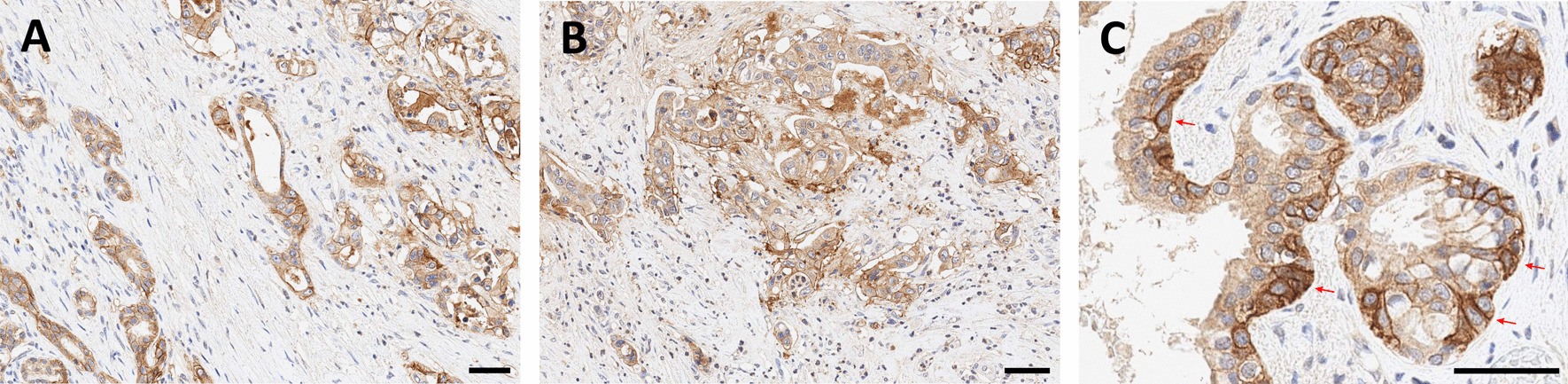
β6-integrin immunohistochemistry of a non-metastatic pancreatic ductal adenocarcinoma (PDAC) localized in the pancreatic tail. Bars indicate 50 µm. The integrin is expressed by most tumor cells (**A**), and a substantial higher expression is observed in the infiltration margin (**B**). Invading tumor cells directly adjacent to the surrounding stroma (examples indicated by arrows) regularly show intense upregulation of β6-integrin expression (**C**). *Note that the β6-subunit dimerizes only with the ubiquitous αv (see Fig. **1**), which is why β6-IHC is indicative for actual αvβ6 expression*

αvβ6-integrin therefore represents an extremely valuable theranostic target, because it potentially enables a precise delineation of carcinoma margins and/or assessment of their invasiveness by molecular (nuclear) imaging, as well as therapeutic intervention with targeted radioligands at the most critical locations. It is found in many carcinomas, such as squamous cell, basal cell, lung adeno, and colon [48], and also in pulmonary fibrosis [49], which expands the potential of αvβ6-targeted imaging beyond oncology. From a clinical perspective, it is important to note that one of the cancers with the worst prognosis, the pancreatic ductal adenocarcinoma (PDAC), has been shown to be most closely associated with αvβ6-integrin, which is found in 88% of primaries, virtually all metastases, and also in its immediate precursor lesions (PanIN3) [50]. Figure 3 shows an exemplary IHC for a non-metastatic PDAC resected from the pancreatic tail. Most of the tumor cells express β6-integrin (A), and in accordance with the proposed biochemical mechanism, a higher density is found in the infiltrative area (B). A frequent feature in PDAC is an upregulation of β6-integrin expression in tumor cells directly adjacent to the surrounding stromal tissue (C), which is consistent with the aforementioned mechanistic considerations. Fibroblasts and other abundant components of the stroma are β6-negative. Addressing αvβ6-integrin thus allows to guide theranostic agents (which includes, but is not limited to, radiopharmaceuticals) to the *tumor cells*, in contrast to other recently emerging carcinoma-targeting agents like FAP inhibitors (referred to as FAPI) which bind to the tumor-associated *fibroblasts* [51]. αvβ6-integrin could thus be a preferred target for all therapeutic schemes which benefit from a specific homing of the respective agents to carcinoma cells, such as targeted drug delivery, or targeted alpha therapy (TAT) in view of the short range of alpha particles (3–4 cell diameters) in tissues.

Although this potential has been known for a long time, αvβ6-integrin as a clinical target has certainly not yet attracted the attention it deserves. Nonetheless, several research groups have made long-term efforts toward lifting this hidden treasure, discovering novel selective αvβ6 ligands [52–55] and transforming them into tracers for single-photon computed emission tomography (SPECT) [56–58] and PET imaging [59–63]. Just recently, some of these radiopharmaceuticals were evaluated in humans for imaging of various carcinomas [64–69] or idiopathic pulmonary fibrosis (IPF) [69–71]. A proof-of-principle could be delivered in all instances, i.e., αvβ6-integrin targeted imaging was shown to be feasible with all agents, for example, of PDAC, head-and-neck squamous cell carcinoma (HNSCC), lung-, mammary-, colon-, and cervical cancer, as well as in IPF. In our opinion, this clearly underscores that a clinical breakthrough of αvβ6-integrin targeted radiopharmaceuticals is only a matter of time.

### αvβ8: The great unknown

The integrin subunit β8 was discovered 30 years ago [72] and is quite similar to β6—it pairs only with αv, the resulting dimer recognizes the RGD sequence, and it is an activator of TGFβ, although by a different mechanism [73]. Contrary to αvβ6, the available data do not obviously point toward a particular clinical application. Although a recent study by Takasaka et al. indicated that various human carcinomas (ovarian, uterine endometrioid, skin, in situ breast ductal, gastric adenocarcinoma, and particularly oral squamous cell carcinoma) contain large fractions of β8 positive tumor cells, the relatively small numbers of investigated specimen (3–22 per entity) call for more detailed investigations [74]. Interestingly, Takasaka and colleagues hypothesize that the αvβ8-integrin expression could be a biomarker for immune checkpoint therapy.

**Fig. 4:**
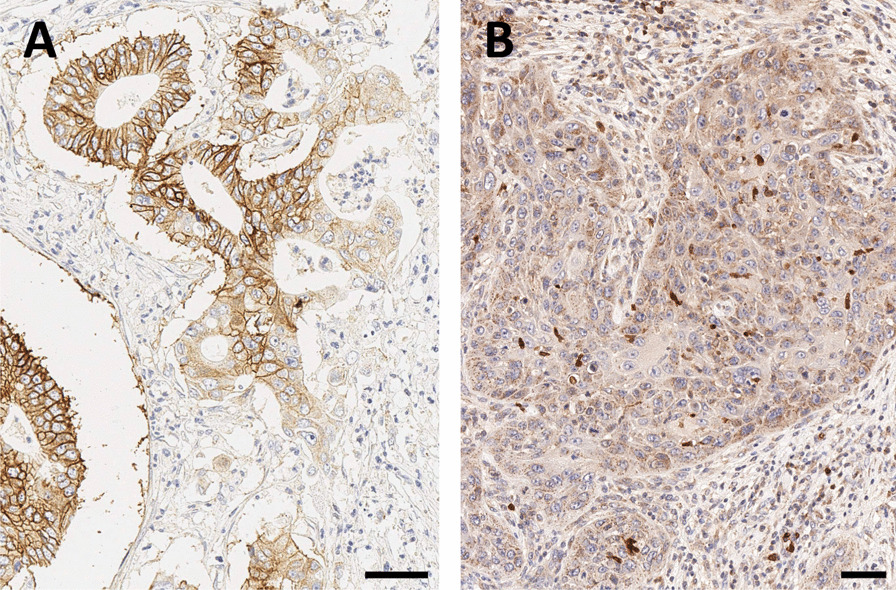
β8-integrin immunohistochemistry of pancreatic ductal adenocarcinoma (PDAC, **A**) and head-and-neck squamous cell carcinoma (HNSCC, **B**). Bars indicate 50 µm. *Note that the β8-subunit dimerizes only with the ubiquitous αv (see Fig. **1**), which is why β8-IHC is indicative for actual αvβ8 expression*

According to our experience, β8-integrin is rarely expressed in human PDAC, but if so, the expression shows a moderate to strong membranous localization in nearly all tumor cells (Fig. 4A). Whether or not this has any clinical implication remains to be elucidated, but we assume that αvβ8-integrin imaging might help in further patient stratification for tailored therapies, or improved prognosis. In human HNSCC, β8-integrin IHC only reveals a slight cytoplasmic positivity of a basal subset of tumor cells. Infiltrative immune cells regularly show a strong β8-integrin expression (Fig. 4B). Further clinical applications in this tumor entity remain to be elucidated as well.

In the past, αvβ8-integrin related discovery was presumably hampered by a lack of selective small-molecule ligands. We would like to remind the reader that the wealth of data and knowledge about αvβ3-integrin is, to a large extent, a result of the early development and wide availability of αvβ3-targeting 'RGD peptides' which, likewise, has not been the case for αvβ8-integrin but now has changed. We believe that the recent development of the selective αvβ8-integrin binding peptide cyclo[GLRGDLp(*N*Me)K] [34] (see Fig. 2) and the corresponding PET imaging agents (see below) will advance the pertinent research.

### α5β1: Angiogenesis, now more than ever!

As outlined above, αvβ3-integrin is not a suitable target to quantify angiogenesis by noninvasive imaging methods. Contrary to that, α5β1-integrin is only poorly expressed on quiescent murine and human endothelial cells [75]. The majority of blood vessels in tumor sections of human colon and breast carcinoma, as well as in subcutaneous xenografts of M21 melanoma cells, are α5β1-integrin positive, while endothelial cells in normal tissue do not express this integrin [76]. This close relation between activation of endothelial cells, angiogenesis, and α5β1-integrin expression underscores the potential of in vivo imaging of angiogenesis using α5β1-targeted radiopharmaceuticals. Despite the ambiguous results obtained with αvβ3 in this context, we strongly advocate to give it another try with α5β1-integrin targeted agents, all the more because a highly potent PET radiopharmaceutical is already available (see below) [27].

**Fig. 5:**
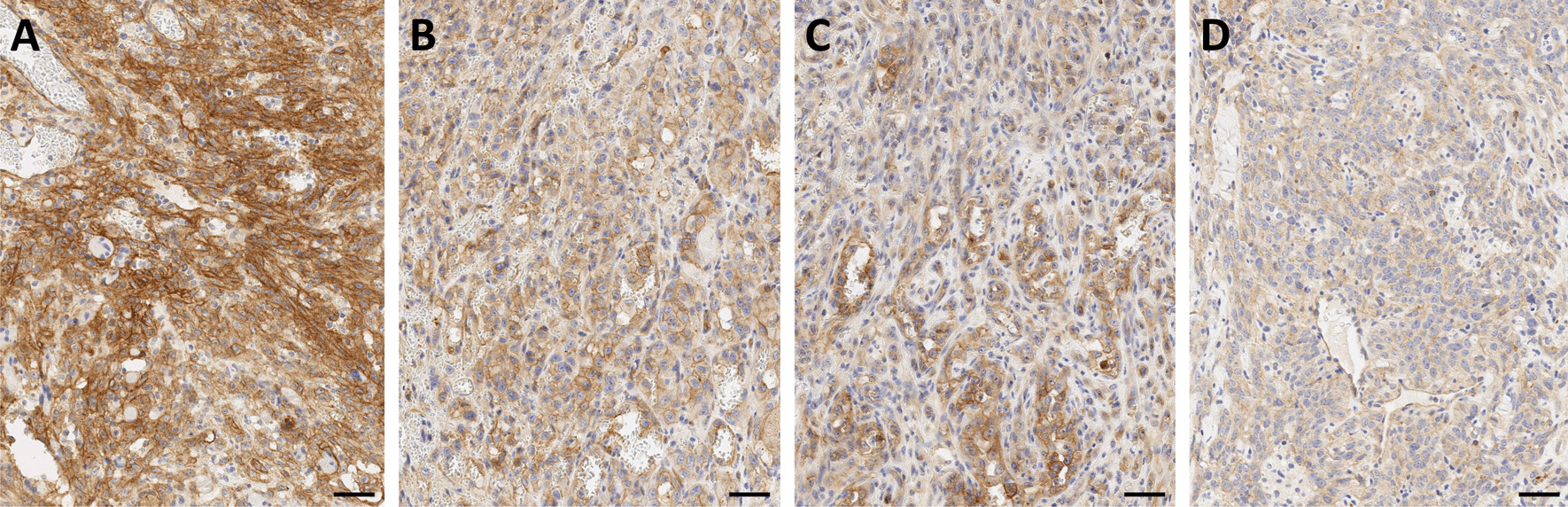
α5-integrin immunohistochemistry of angiosarcoma. A high-grade (**A**), or a homogeneous (**B**) or heterogeneous (**C**) medium-grade expression was observed in 11 out of 12 specimens, and a weak expression in one (**D**). *Note that the α5-subunit dimerizes only with the ubiquitous β1 (see Fig. **1**), which is why α5-IHC is indicative for actual α5β1 expression*

We furthermore hypothesized that α5β1-integrin could also be overexpressed by tumors of vascular origin. α5-IHC of a small cohort of 12 human angiosarcomas from different body sites indeed revealed a strong to medium α5-expression in 11 out of 12 specimens, resulting in a very encouraging incidence of > 90% (see Fig. 5). We therefore envisage a potential field of application for clinico-radiological confirmation of angiosarcoma vs. its differential diagnoses.

## Multimers of integrin ligands

In view of the popularity of cyclic pentapeptides of the cyclo[RGDxK] (x = y, f) type, it is hard to imagine that a greater variety of conjugates has been generated for any other small-molecule targeting motif. The same might apply to multimers thereof [2]. The impact of multiplicity has been evaluated for c[RGDxK]'s in several systematic studies, which invariantly showed that a higher degree of multiplicity increased the affinity of the constructs [78–85] and frequently resulted in improved in vivo targeting properties, i.e., higher target-specific uptake [86, 87].

### ^68^ Ga-labeled trimers based on the Triazacyclononane-triphosphinate (TRAP) chelator core

**Fig. 6:**
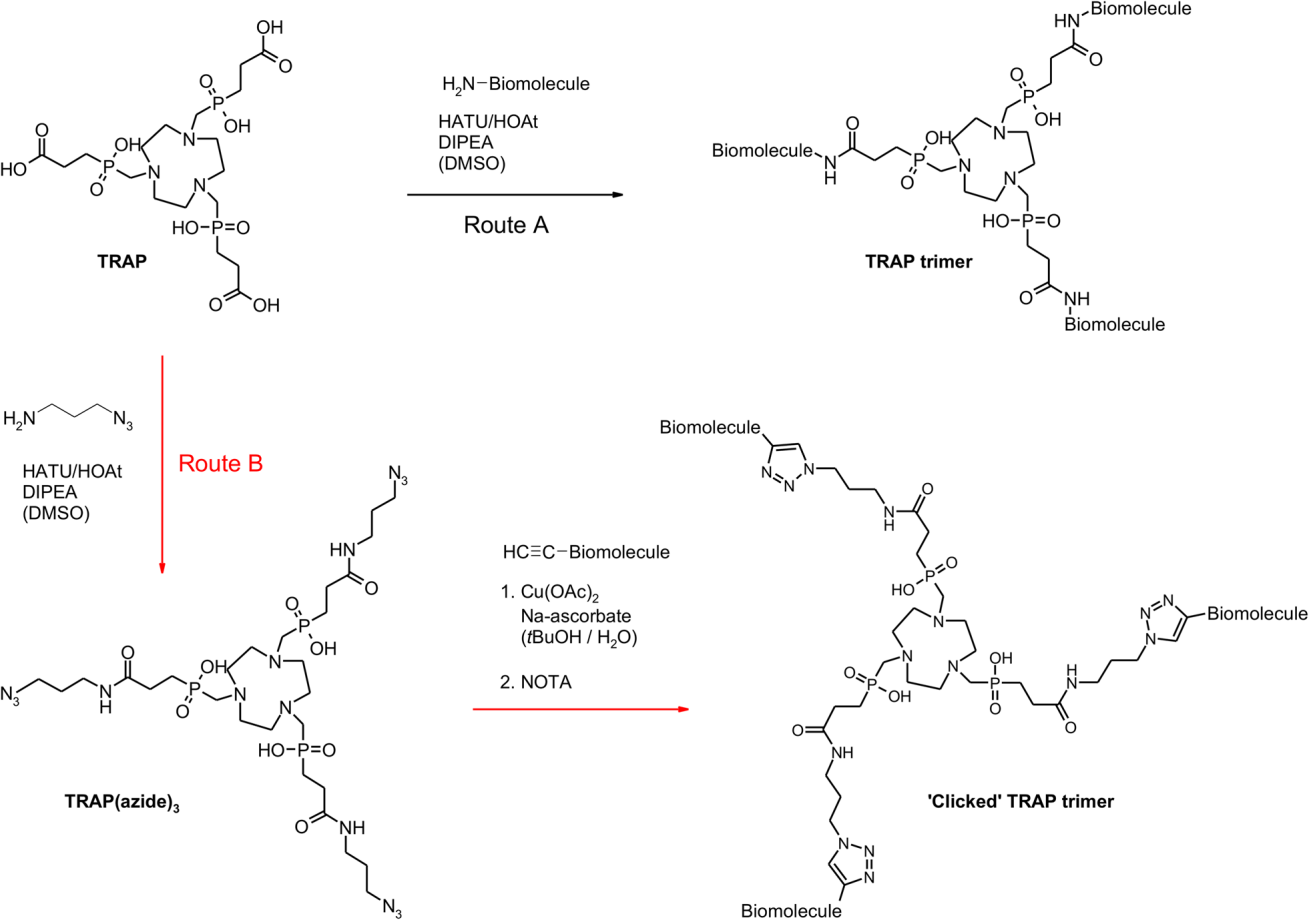
Synthesis of trimeric TRAP bioconjugates. The preferred route **B** for elaboration of trimeric integrin ligands by means of 'click chemistry' (CuAAC) is indicated by red arrows [91]. Of note, the CuAAC coupling was occasionally conducted with azide-decorated integrin ligands and alkyne-functionalized TRAP (obtained by amide coupling of propargyl amine, H_2_N–CH_2_–C≡CH, in step 1), e.g., for synthesis of ^68^ Ga-Aquibeprin (see Fig. 7)

In the framework of the German Research Foundation's Collaborative Research Centre 824, we pursued a unique approach toward multimeric integrin ligands for application in nuclear imaging. We utilized the ^68^ Ga-chelator TRAP (1,4,7-triazacyclononane-1,4,7-tris[methylene-(2-carboxyethylphosphinic acid)], which was originally developed at Charles University in Prague in the late 2000's [87], to generate trimeric conjugates whose three conjugated peptides are connected to the chelator core in an identical fashion owing to the system's formal molecular C_3_ symmetry [88]. TRAP (the acronym referring to **tr**iaz**a**cyclononane-tri**p**hosphinate) bears three chemically equivalent carboxylic acid moieties which are not involved into radiometal complexation and, therefore, can be functionalized by amide formation with a large variety of biologically active compounds comprising primary amines [90, 91]. Elongation of the carboxylate conjugation handles with short linkers, bearing terminal alkynes or azides on the other end, paved the way to a more convenient conjugation protocol, employing copper(I)-mediated [91] or strain-promoted [92] alkyne–azide cycloaddition (commonly referred to as the archetype of 'click chemistry'). This approach has the obvious advantage that even biomolecules comprising chemical groups that could interfere with peptide-coupling conditions (amines, carboxylic acids, alcohols, phenols, guanidines, and others) do not have to be equipped with protecting groups [93], thus facilitating the rapid synthesis of a variety of trimeric ligands for biological evaluation (Fig. 6).

TRAP furthermore provides the advantage of exceptionally efficient gallium(III) complexation [94], which enables ^68^ Ga-labeling of the respective trimers with unparalleled molar activity [95]. It tolerates comparably high concentrations of frequently occurring metal ion contaminants in ^68^Ge/^68^ Ga generator eluates and ^68^ Ga labeling solutions, such as Fe^III^ [96], Zn^II^, and Cu^II^ [97], giving rise to very robust labeling protocols and a reliable supply of radiopharmaceuticals. Altogether, the TRAP technology represents a convenient and straightforward route toward symmetrical ^68^ Ga-labeled integrin ligand trimers, which enabled us to investigate the effect of integrin ligand multimerization in a systematic fashion.

### From monomers to trimers: patterns of enhanced performance

**Fig. 7:**
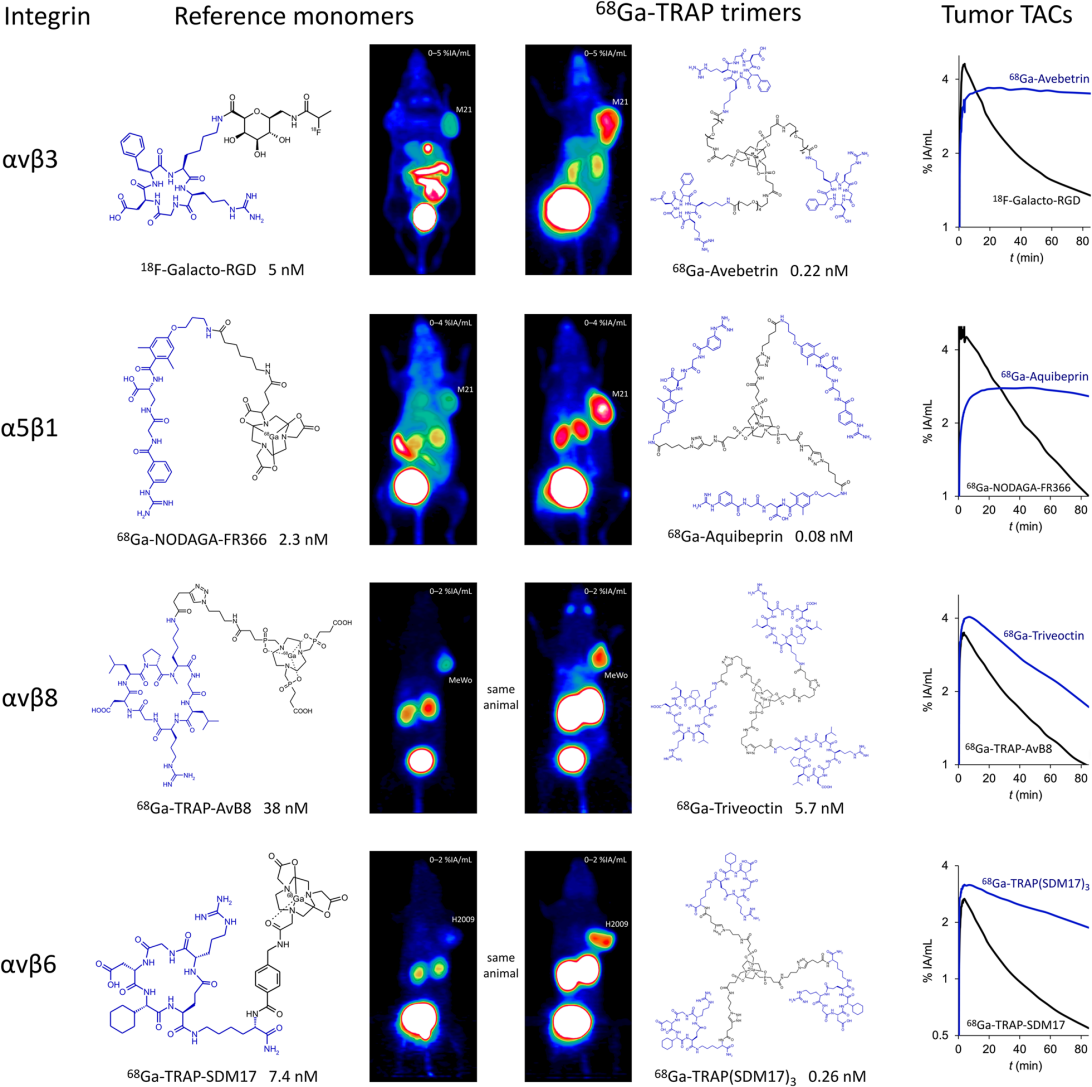
Comparison of µPET images (MIPs of static scans; αvβ3, α5β1, αvβ6: 75 min p.i.; αvβ8: 60 min p.i.) in SCID mice bearing subcutaneous xenografts of integrin-expressing tumor cell lines (M21: αvβ3 and α5β1; MeWo: αvβ8; H2009: αvβ6) for the radiolabeled monomers ^18^F-Galacto-RGD [83], ^68^ Ga-NODAGA-FR366 [102], ^68^ Ga-TRAP-AvB8 [34], and ^68^ Ga-TRAP-SDM17 [32], with their corresponding TRAP trimers ^68^ Ga-Avebetrin [27, 83], ^68^ Ga-Aquibeprin [27], ^68^ Ga-Triveoctin [104], and ^68^ Ga-TRAP(SDM17)_3_ [105], respectively. Structures of comprised integrin ligands (see Fig. 2) are highlighted in blue. *IC*_50_ values (given in nM) are denoted for the respective targeted integrins (see left column). Time-activity curves in the right column were derived from ROI-based analysis of 90-min dynamic µPET scans (the high initial uptakes for ^18^F-Galacto-RGD and ^68^ Ga-NODAGA-FR366 are signal crosstalk artifacts resulting from non-optimal tumor position close to the main vein)

During the entire 12-year term of CRC824, we systematically investigated the properties of trimeric integrin ligands in order to identify regular patterns of affinity enhancement and altered in vivo performance upon switching from monomers to multimers (see Fig. 7). Building on the achievements made with ^18^F-Galacto-RGD in the early 2000's [3–5, 99, 100], we first investigated a series of c[RGDfK] trimers [83] and chose a PEG_4_-linked conjugate because it showed the best affinity (initially referred to as ^68^ Ga-TRAP(RGD)_3_, but later renamed to ^68^ Ga-Avebetrin for typographic simplicity, allowing for a more consistent transfer into abstract databases and other repositories) [27, 100]. Its nearly 23-times higher αvβ3-integrin affinity compared to ^18^F-Galacto-RGD resulted in an improved delineation of αvβ3-expressing M21 tumors in µPET because of a drastically enhanced tumor retention (Fig. 7). We also investigated ^68^ Ga-NODAGA-c[RGDyK] in the same setting and found that its in vivo properties were nearly identical to ^18^F-Galacto-RGD [101], confirming that the observed superiority of the multimer is likely to apply in comparison with any RGD monomer [83].

A similar improvement of integrin affinity (≈ 26-fold), PET imaging performance, and tumor retention was observed upon trimerization of the α5β1-selective peptidomimetic FR366, as shown by comparison of data for ^68^ Ga-NODAGA-FR366 [102] and the trimer ^68^ Ga-Aquibeprin [27, 100] (Fig. 7). The latter was assembled by means of click-chemistry due to serious issues with protecting group chemistry which made trimerization by amide coupling a cumbersome endeavor. The overall simplicity and almost quantitative coupling yields prompted us to employ this protocol (see Fig. 6, route B) for all further work with TRAP. Of note, ^68^ Ga-Avebetrin and ^68^ Ga-Aquibeprin turned out to be a nearly perfect complementary pair of tracers for αvβ3- and α5β1-integrin. Their virtually identical biokinetics but opposite selectivities for the two addressed integrin subtypes (*IC*_50_ for αvβ3 and α5β1: ^68^ Ga-Avebetrin: 0.22 and 39 nM; ^68^ Ga-Aquibeprin: 620 and 0.08 nM) allowed for independent mapping of the two angiogenesis-related endothelial integrins that were simultaneously expressed by M21 tumors [27]. ^68^ Ga-Aquibeprin furthermore enabled the sensitive imaging of arthritic joints in collagen-induced arthritis (CIA) rats even before the onset of clinical symptoms (swelling, redness), which interestingly did not rely on angiogenesis-related expression but on a high α5β1-integrin density on the proliferating cartilage surface [103].

The same pattern of affinity enhancement, increased tumor uptake, and prolonged tumor retention was also observed for αvβ8- and αvβ6-integrin binding peptides upon trimerization, which yielded the radiopharmaceuticals ^68^ Ga-Triveoctin [104] and ^68^ Ga-TRAP(SDM17)_3_ [105]. Figure 7 shows that although the effect is less pronounced than for the αvβ3- and α5β1-integrin ligands, a substantial gain of image quality is nonetheless achieved. Whether the concomitant increase in kidney retention is related to increased molecular size or unspecific uptake in renal tubular cells requires further investigation.

## Clinical translation of ^68^ Ga-labeled trimeric integrin ligands

**Fig. 8:**
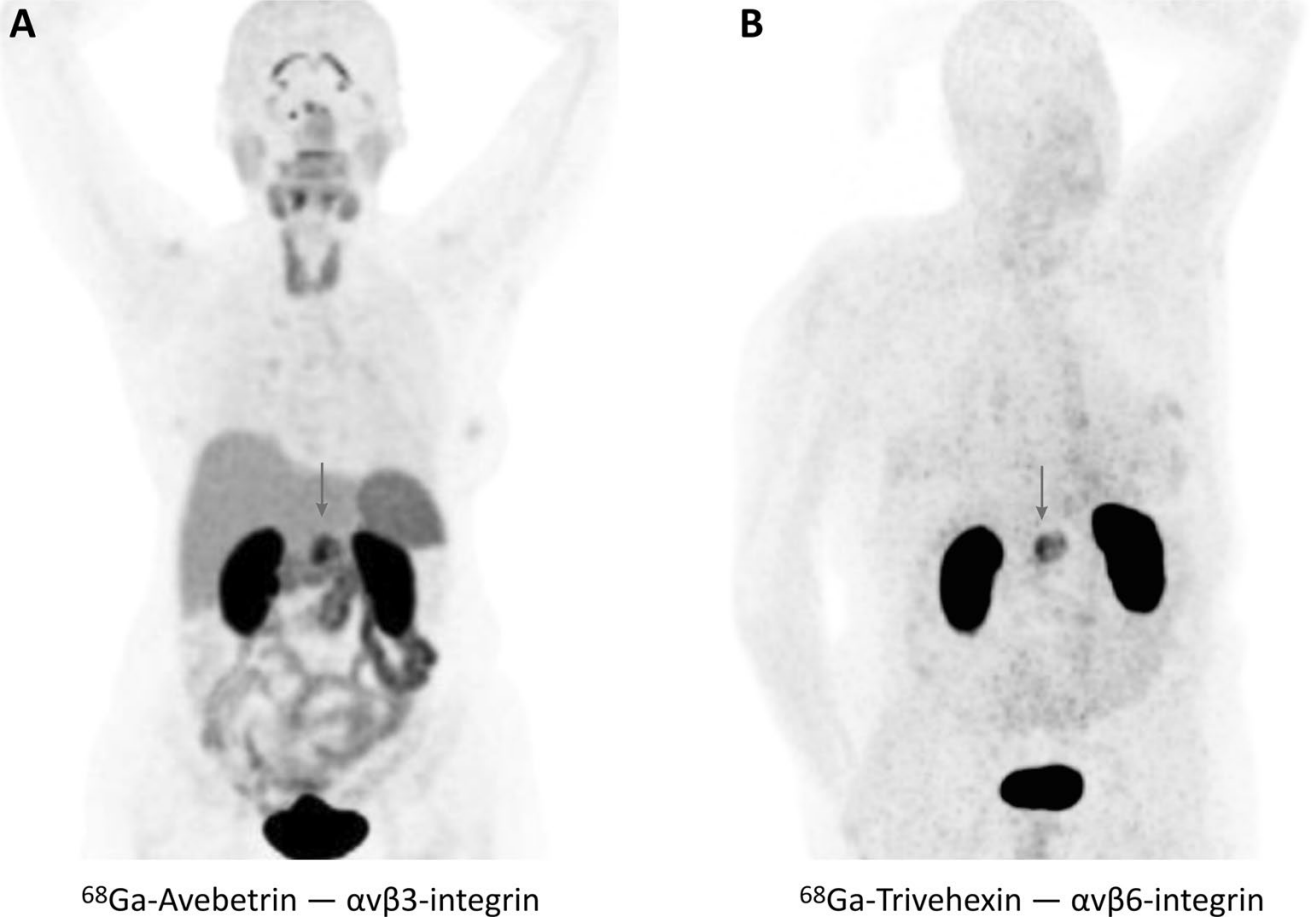
Imaging of pancreatic cancer in humans (tumor positions indicated by arrows). **A**: ^68^ Ga-Avebetrin PET (144 MBq, 46 min p.i.) of a female patient with poorly differentiated PDAC, showing focal uptake in the tumor (SUV_max_ = 8.5) [106]. The uptake pattern in the organs is comparable to other αvβ3-integrin tracers [6] and most likely originates in physiological αvβ3-integrin expression. **B**: ^68^ Ga-Trivehexin PET (87 MBq, 70 min p.i.) of a male patient with histologically proven PDAC (SUV_max_ = 13.1) [109]. Apart from excretion-related activity in the kidneys, no significant organ uptakes are observed, which is in accordance with a generally low expression of αvβ6-integrin in adult human tissues

Although the discussed molecular design strategies yielded convincing results in rodent models, it is all but obvious that the enhanced performance of the trimeric radiopeptides or -peptidomimetics actually translates to a higher diagnostic value in a clinical setting [2]. αvβ3-integrin PET with ^68^ Ga-Avebetrin nonetheless showed a good image contrast and enabled, for example, the localization of a PDAC lesion (Fig. 8A) [106]. However, we also observed a prominent physiological uptake pattern that was quite similar to other radiopharmaceuticals addressing the same target, including ^18^F- and ^68^ Ga-labeled c[RGDxK] monomers [6]. The clinical value of ^68^ Ga-Avebetrin and other αvβ3-integrin tracers thus appears to be largely independent from the molecular design and, as discussed above, is always limited by organ uptake patterns which presumably originate in physiological αvβ3-integrin expression.

A different situation is encountered for αvβ6-integrin targeted radiopharmaceuticals due to the generally low level of αvβ6 expression in adult human tissues [39]. All αvβ6-integrin PET tracers that were hitherto tested in humans nevertheless showed substantial non-specific organ uptakes, particularly in the gastrointestinal tract [64–68, 68], which could complicate the interpretation of images in these areas. Based on our encouraging results with other integrin ligands (see Fig. 7), we also synthesized a trimer of the highly selective cyclic nonapeptide c[FRGDLAFp(*N*Me)K] [33], which, however, showed far too high non-specific organ uptake in mice [107]. Trimerization of a slightly modified version of the same peptide, comprising tyrosines instead of phenylalanines, finally resulted in a more suitable radiopharmaceutical named ^68^ Ga-Trivehexin. Its favorable preclinical data encouraged a clinical translation for imaging of head-and-neck cancers as well as metastatic pancreatic ductal adenocarcinoma [108]. Figure 8B shows that apart from excretion-related kidney uptake, ^68^ Ga-Trivehexin only accumulated in a PDAC lesion [109]. A comparison of αvβ3- and αvβ6-integrin PET of PDAC patients, obtained with ^68^ Ga-Avebetrin and ^68^ Ga-Trivehexin, clearly demonstrates that radiopharmaceuticals targeting αvβ6-integrin could indeed possess a higher theranostic value (Fig. 8). The fact that the standard PET tracer [^18^F]FDG is not suitable for diagnosis of pancreatic cancer in its early stages [110] underscores the relevance of ^68^ Ga-Trivehexin for PET imaging of PDAC and might open new avenues for planning of surgery and monitoring of chemotherapies.

## Conclusions

The functional diversity of the 24 different integrins bears a huge, largely untapped potential for novel theranostic approaches, particularly in the field of nuclear medicine. In this review, we outlined some lines of thought on how this hidden treasure could be lifted in the future, driven by novel, selective ligands, and optimized radiopharmaceuticals. We described that multimers of integrin ligands often display superior performance at the preclinical stage and furthermore demonstrated that a ^68^ Ga-labeled trimeric αvβ6-integrin-targeted PET radiopharmaceutical shows excellent performance for imaging of pancreatic carcinoma in a clinical setting. Hence, we believe that multimeric probes in molecular imaging are no longer a future vision but, from now on, should be considered clinical reality [2]. We are furthermore convinced that tracers for integrins other than αvβ3—first and foremost, for αvβ6—will define the future of integrin imaging and re-shape the general perception of integrins as theranostic targets.

## Data Availability

The graphs and images in the current review are reproduced or rearranged on the basis of previously published data; all original publications are referenced at the respective positions. Exemplary, previously unpublished IHC data in Figs. 3, 4, 5 are included completely in this article.
